# Reaching the poor with health interventions: programme-incidence analysis of seven randomised trials of women's groups to reduce newborn mortality in Asia and Africa

**DOI:** 10.1136/jech-2014-204685

**Published:** 2015-08-05

**Authors:** Tanja A J Houweling, Joanna Morrison, Glyn Alcock, Kishwar Azad, Sushmita Das, Munir Hossen, Abdul Kuddus, Sonia Lewycka, Caspar W Looman, Bharat Budhathoki Magar, Dharma S Manandhar, Mahfuza Akter, Albert Lazarous Nkhata Dube, Shibanand Rath, Naomi Saville, Aman Sen, Prasanta Tripathy, Anthony Costello

**Affiliations:** 1 Institute for Global Health, University College London, London, UK; 2 Department of Public Health, Erasmus MC University Medical Center Rotterdam, Rotterdam, The Netherlands; 3 Perinatal Care Project (PCP), Diabetic Association of Bangladesh (BADAS), Dhaka, Bangladesh; 4 Society for Nutrition, Education and Health Action (SNEHA), Urban Health Centre, Shahunagar, Dharavi, Mumbai, Maharashtra, India; 5 Mother and Infant Research Activities (MIRA), Kathmandu, Nepal; 6 MaiMwana Project, Mchinji, Malawi; 7 Ekjut, Chakradharpur, Jharkhand, India

**Keywords:** Health inequalities, INTERNATIONAL HLTH, HEALTH BEHAVIOUR, NEONATAL, REPRODUCTIVE HEALTH

## Abstract

**Background:**

Efforts to end preventable newborn deaths will fail if the poor are not reached with effective interventions. To understand what works to reach vulnerable groups, we describe and explain the uptake of a highly effective community-based newborn health intervention across social strata in Asia and Africa.

**Methods:**

We conducted a secondary analysis of seven randomised trials of participatory women's groups to reduce newborn mortality in India, Bangladesh, Nepal and Malawi. We analysed data on 70 574 pregnancies. Socioeconomic and sociodemographic differences in group attendance were tested using logistic regression. Qualitative data were collected at each trial site (225 focus groups, 20 interviews) to understand our results.

**Results:**

Socioeconomic differences in women's group attendance were small, except for occasional lower attendance by elites. Sociodemographic differences were large, with lower attendance by young primigravid women in African as well as in South Asian sites. The intervention was considered relevant and interesting to all socioeconomic groups. Local facilitators ensured inclusion of poorer women. Embarrassment and family constraints on movement outside the home restricted attendance among primigravid women. Reproductive health discussions were perceived as inappropriate for them.

**Conclusions:**

Community-based women's groups can help to reach every newborn with effective interventions. Equitable intervention uptake is enhanced when facilitators actively encourage all women to attend, organise meetings at the participants’ convenience and use approaches that are easily understandable for the less educated. Focused efforts to include primigravid women are necessary, working with families and communities to decrease social taboos.

## Introduction

The Every Newborn Action Plan (ENAP), formally launched in South Africa in June–July 2014, envisages a world without preventable newborn deaths. Laudable as the plan is, efforts under ENAP will fail if the poor are not reached. Inequalities in maternal and child survival in low-income and middle-income countries (LMICs) are very large, and progress towards Millennium Development Goals 4 and 5 has been uneven.^1–5^ One-third of global childhood deaths are attributable to mortality inequalities within countries.^1^ Effective interventions exist,^6^ ^7^ but rarely reach the most in need.^8–15^ Even uptake of ‘simple’ interventions considered to be propoor, such as immunisation, is usually higher among the better-off.^8–10^

Little is known about what works to effectively reach the most in need.^16^ Understanding why interventions do not reach poor and otherwise vulnerable groups, and how to remedy this, requires a combination of programme-incidence analysis, which “determine[s] the distribution of programme outputs across socioeconomic groups within the population that it serves”,^11^ and a qualitative understanding of the barriers to and facilitators of intervention uptake. Unfortunately, such research is uncommon.^16^

Community-based interventions to improve newborn survival are becoming increasingly popular.^17^ ^18^ Yet, by engaging whole communities without explicitly targeting lower strata, they run the risk of elite capture, in which the locally powerful influence interventions to their benefit.^19^ There are indeed indications that community-based interventions can reinforce existing social hierarchies.^20–24^ Trials of community-based interventions with participatory women's groups to improve maternal and newborn health in Asia and Africa provide an opportunity to gather evidence on how to address the exclusion of lower social strata from health interventions. A recent meta-analysis showed that women's groups can significantly reduce newborn mortality.^25^ It remains unknown whether they reached the most in need—poorer and less-educated women—and young women in their first pregnancies whose newborns are at higher risk.

This study describes socioeconomic and sociodemographic inequalities in the uptake of this highly effective intervention to reduce newborn mortality in Asia and Africa, using data from seven trials, and explains what works to reduce inequalities, using new qualitative data collection.

## Methods

### Setting

The seven cluster randomised trials of community-based women's group interventions were conducted in an urban site (Mumbai, India) and in rural sites in India, Nepal, Bangladesh and Malawi.^25–33^ The trials were similar in structure and content.^25^ Women's groups met every month (fortnightly in Mumbai), under the guidance of a local facilitator, for a 2-year to 4-year period. The facilitator guided the groups through a participatory learning and action cycle, in which they identified and prioritised problems during pregnancy, delivery and the newborn period, developed and implemented strategies to address them together with other community members, and then evaluated their strategies. While the groups were generally open to everyone and included community outreach activities, the primary target group was women aged 15–49 years, especially newly married (in the Asian sites) and/or pregnant women.

### Quantitative analysis of trial data

Surveillance systems followed up all births during the trials, prospectively. Women were interviewed at around 6 weeks post partum, with questions on social position (SP) and women's group attendance. We restricted our analyses to permanent residents of the trials’ intervention arms, including all detected pregnancies, irrespective of birth outcome.

Attendance at the women's groups was our main outcome. Questions on attendance varied somewhat between the sites, some asking about current attendance or membership and others about whether women had ever attended. We expect this to only influence estimates of overall, and not differentials in, attendance, and attendance levels were more strongly influenced by the population coverage of women's groups than by the question asked.^25^

Attendance was examined in two dimensions of social position: socioeconomic and sociodemographic. Socioeconomic position was measured using maternal education (never went to school, 1–4, 5–10, 11+ years schooling) and household economic status (wealth quintiles based on a principal components analysis-based asset index). Sociodemographic position is potentially an important determinant of participation in community-based interventions. In many South Asian settings, young women and women who are pregnant with their first baby have a lower SP and less decision-making power in the household and community than older women who already have children.^34^ We considered two variables: age (<20, 20–24, 25–29, 30–34 and 35+ years) and gravidity (primigravid vs multigravid). For advanced pregnancies, which either resulted in a birth or in a maternal death prior to delivery, we asked if this was the first delivery or first pregnancy (site dependent).

#### Statistical analyses

All analyses were adjusted for the trials’ cluster design using random-effects logistic regression. Analyses were run for each trial separately. For trials with relatively high and often increasing attendance, we examined time trends using the models below. For the trials with attendance at 1–3%, we analysed all years combined. We hypothesised that the intervention systematically reached higher social strata first, that is, in year 1 or when overall levels were low.

First, cluster adjusted attendance (%) was estimated per year using logistic regression models for the total population and by SP. For individual j in cluster i:

For the total population:
1
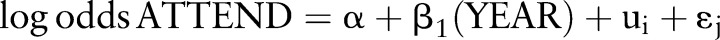
where the βs for YEAR are the change in attendance between subsequent intervention years compared with year 1 and u is the random-effect term. Since year was treated as categorical variable, β_1_ represents a vector of values with the length of the number of study years minus the baseline year.

By social position:
2
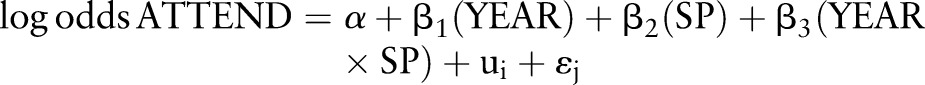
where the βs for YEAR are the change in attendance between subsequent years compared with year 1 for the lowest SP group (reference), the β for SP is the effect of SP in year 1, and the βs for the interaction term YEAR×SP the difference in change between years for lower and higher SP groups. Again, since year and SP were treated as categorical variables, β_1_, β_2_ and β_3_ represent a vector of values.

Socioeconomic and sociodemographic inequalities in attendance were estimated for each year, using a reparameterisation of equation 2:3
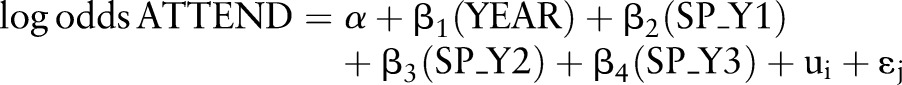
where SP_Y1, SP_Y2 and SP_Y3 are the effects of SP in years 1, 2 and 3 respectively. Inequalities were expressed in ORs.

A combined Wald test was used to test the null hypothesis that the overall effect of SP on attendance was equal to 1 (no effect). As the pattern of attendance may change over time, the Wald test was performed for each year separately. A combined Wald test was used to test if the effect of SP changed across years.

Second, we examined whether observed socioeconomic and sociodemographic inequalities in attendance could be explained by each other, by adding each of the measures of SP one-by-one to the reparameterised versions of equation 2, thus creating separate models for each measure of SP adjusted for one other measure of SP. The reliability of the main random-effect models was assessed with quadrature checks. All analyses were run using Stata12 (Stata, College Station, Texas, USA).

### Qualitative data collection and analysis

We used purposive sampling frameworks to explore questions arising from the above analyses. In three or four clusters per trial site, we conducted 24–38 focus group discussions (FGDs), with women who had or had not attended groups, sampling on the basis of (1) age and parity, (2) education and economic status and (3) ethnicity/caste (for which findings will be presented in a separate paper; table 1). In the same clusters, we also conducted FGDs with women's group facilitators and family decisionmakers, and an interview with a community key informant. In total, 225 FGDs, 2 semistructured interviews and 18 key informant interviews were conducted. We used trial surveillance databases, local field staff and community health volunteers to locate participants.

**Table 1:** Qualitative data collection overview

		Trial site					
Respondent category	A or NA	Mumbai, urban India	Bangladesh	Rural India	Nepal-Makwanpur	Nepal-Dhanusha	Malawi
*FGDs with women who delivered a baby*							
Sociodemographic position							
Young and primigravid	A	3	3	3	3 (1 SSI, 2 FGDs)	3	2
	NA	3	3	3	3 (1 SSI, 2 FGDs)	3	2
Not primigravid and not young	A	3	3	3	3	3	2
	NA	3	3	3	3	3	2
Socioeconomic position							
Low (no education, poorest quintile)	A	2	3	3	3	3	2
	NA	3	3	3	3	3	2
High (some education, quintile 2–5)*	A	2	–	–	3	3	–
	NA	3	–	–	3	3	–
Medium socioeconomic position*	A	–	3	3	–	–	2
	NA	–	3	3	–	–	2
High (11+ year education, richest quintile)*	A	–	3	1	–	–	1
	NA	–	3	1	–	–	1
Sociocultural position							
Low caste	A	–	–	3	3	3	–
	NA	–	–	3	3	3	–
Not low caste	A	–	–	3	3	3	–
	NA	–	–	3	3	3	–
							
Hills ethnicity	A	–	–	–	–	3	–
	NA	–	–	–	–	3	–
Non-hills ethnicity	A	–	–	–	–	3	–
	NA	–	–	–	–	3	–
*FGDs and KIIs with key informants*							
*FGDs*							
Decisionmaker	A	3	3	3	3	3	3
Women’ s group facilitator		2	3	3	3	3	3
*KIIs*							
Community people		3	3	3	3	3	3
Total data points							
Total FGDs: 225 Total KIIs: 18		**30** (27 FGDs, 3 KIIs)	**39** (36 FGDs, 3 KIIs)	**47** (44 FGDs, 3 KIIs)	**45** (40 FGDs, 3 KIIs, 2 SIIs)	**57** (54 FGDs, 3 KIIs)	**27** (24 FGDs, 3 KIIs)
Total SSIs: 2							

*For the sampling in Nepal-Makwanpur, Nepal-Dhanusha and Mumbai, it was only possible to distinguish two socioeconomic categories (low and high), while for Bangladesh, rural India and Malawi it was possible to also make a distinction between medium socioeconomic position and the elite (11+ years of education, richest quintile).

A, Attenders; FGDs, focus group discussions; KIIs, Key informant interviews, NA, not applicable; SSI, semistructure interview.

Topic guides were developed for each trial site and adapted after field testing. Data were collected in local languages by experienced qualitative researchers, after verbal consent. Data were recorded, transcribed and analysed at each site according to a common coding structure developed with the researchers across all sites. Emergent codes were added where necessary. Following the framework approach to analysis,^35^ researchers compared tabulated responses between participants and clusters. They wrote detailed descriptions of each code in English using illustrative quotes. The descriptions and three to five transcripts that best represented the data were translated into English and read by the principal qualitative investigator to consolidate findings.

Ethical approval for the study was obtained from the UCL Research Ethics committee (project ID 3229/001) and local research ethics committees.

## Results

### Quantitative findings

The proportion of women with no education varied substantially between the sites, from 20% to 30% to around 75% (table 2). The socioeconomic elite (11+ years of education and/or richest group) segment was very small everywhere. Women's group attendance varied considerably between the trials. Among women who had recently delivered a baby, 1–3% had attended a group in Mumbai and the first Bangladesh trial; attendance elsewhere was 10–55% (table 3). Socioeconomic differences in attendance were usually small (tables 4–8). When they were large in absolute and relative terms, attendance was lower among the elite (tables 3–8). Where attendance favoured the rich, this was in year 1 only (rural India), with later years favouring poorer/middle socioeconomic strata. Sociodemographic position was a strong determinant of attendance everywhere except Makwanpur, Nepal, with much lower attendance among young (<20 years) and primigravid women. The effects of sociodemographic position appeared stronger in the African than in the South Asian trials.

**Table 2:** Distribution of pregnancies by socioeconomic position, seven trials, all intervention years combined

Trial	Mumbai, urban India trial		1st Bangladesh trial¶		2nd Bangladesh trial¶		Rural India trial		Nepal-Makwanpur trial		Nepal-Dhanusha trial		Malawi trial	
Trial location (implementing organisation)	Slum areas in Mumbai, India (SNEHA)		3 districts in rural Bangladesh (PCP)				Jharkhand and Odisha state (Ekjut)		Rural Nepal, Makwanpur district (MIRA)		Rural Nepal, Dhanusha district (MIRA)		Rural Malawi, Mchinji district (MaiMwana)	
	n	Per cent	n	Per cent	n	Per cent	n	Per cent	n	Per cent	n	Per cent	n	Per cent
Total	5996	100	15 537	100	11 064	100	9594	100	2969	100	13 904	100	11 510	100
Maternal education*, years schooling														
Never went to school	1457	24	5018	32	2220	20	7186	75	2209	75	10 339	75	2115	19
1–4	323	5	5420	35	4149	38	500	5	307	10	1636	12	3920	34
5–10	3601	60	4653	30	4361	39	1741	18	447	15	1056	8	5014	44
11+†	615	10	446	3	334	3	167	2	–	–	773	6	315	3
Household economic status‡														
Poorest	1235	21	7626	49	2556	23	2286	24	1513	51	3128	23	2978	26
Next-poor	1175	20	2079	13	2398	22	4508	47	929	31	2800	20	1171	10
Middle§	1312	22	2056	13	2052	19	1174	12	–	–	2744	20	2428	21
Next rich	1242	21	1969	13	2346	21	880	9	265	9	2473	18	4423	39
Rich	1032	17	1807	12	1711	15	746	8	246	8	2753	20	483	4
Maternal age, years														
<20	492	8	2704	17	1862	17	1110	12	186	6	3115	22	1474	13
20–24	2792	47	5778	37	4131	37	3037	34	1061	36	5396	39	3667	32
25–29	1942	32	4167	27	2987	27	2683	30	802	27	3657	26	2716	24
30–34	595	10	1833	12	1464	13	1429	16	471	16	1192	9	1913	17
35+	169	3	1035	7	618	6	717	8	443	15	544	4	1613	14
Gravidity														
First pregnancy	1935	32	5280	34	3543	32	2556	27	408	14	4489	32	2001	18
Not first pregnancy	4061	68	10 257	66	7519	68	7030	73	2557	86	9415	68	9334	82

The percentage of respondents with missing values for the variables of interest was very low, between 0 and 2% in each of the sites, with the exception of 6% missing values for maternal age in the rural India trial and 14% missing values for attendance in the Malawi trial.

*Data on maternal education were available by level and number of years of education for all trials except the one in rural India and the first Bangladesh trial. Categories were constructed such that they were as similar as possible across the sites: no education; 1–4 years of education (1–3 years for rural India and 1–5 for Bangladesh); 5–10 years of education (4–10 for rural India, 6–10 for Bangladesh); 11+ years of education.

†For Nepal-Makwanpur, there were only seven women with 11+ years of schooling. These were merged with the 5–10 years of schooling category.

‡We made as equally sized wealth groups (quintiles) in each site as possible, but in some sites there remained considerable population heaping in some wealth categories.

§For Nepal-Makwanpur, the questionnaire included four predefined asset levels; the middle category was therefore excluded.

¶The first and second Bangladesh trial were conducted in the same study areas. The second trial was set up to test the hypothesis that the lack of intervention effect in the first trial was due to the very low coverage of the intervention in the study population.

MIRA, Mother and Infant Research Activities; NA, not applicable; PCP, Perinatal Care Project; SNEHA, Society for Nutrition, Education and Health Action.

**Table 3:** Women's group attendance by socioeconomic and sociodemographic position (%) per trial and intervention year

	Mumbai, urban India trial	1st Bangladesh trial	2nd Bangladesh trial			Rural India trial			Nepal-Makwanpur trial		Nepal-Dhanusha trial				Malawi trial			
Trial	Years 1–3	Years 1–3	Year 1	Year 2	Year 3	Year 1	Year 2	Year 3	Year 1	Year 2	Year 1	Year 2	Year 3	Year 4	Year 1	Year 2	Year 3	Year 4
Total	1	3	31	34	38	14	36	54	39	37	9	10	14	14	50	52	55	57
Maternal education, years schooling																		
Never went to school	1	3	33	38	40	13	36	55	38	37	9	10	13	14	48	52	59	60
1–4	2	3	35	37	43	17	34	60	39	39	9	11	15	16	53	54	57	58
5–10	1	2	27	32	33	15	35	53	44	40	10	12	16	14	51	52	52	55
11+	1	2	11	19	17	15	29	29	–	–	12	11	15	13	35	49	43	50
Household economic status																		
Poorest	1	3	36	38	44	10	32	52	38	34	11	11	13	16	47	49	55	55
Next poor	2	3	30	35	38	14	39	58	39	41	11	11	13	14	51	54	57	56
Middle	2	3	30	37	36	11	36	61	–	–	7	11	14	15	53	54	56	59
Next rich	1	3	28	34	41	20	33	52	45	39	8	10	16	16	51	54	54	58
Rich	1	2	24	26	29	15	34	32	34	39	6	8	13	11	42	49	51	43
Maternal age, years																		
<20	1	2	25	26	32	10	25	43	31	29	7	7	10	6	39	30	29	32
20–24	1	2	29	33	37	13	31	52	42	36	9	10	12	12	49	52	52	52
25–29	2	4	34	39	39	18	41	57	38	45	13	13	16	19	55	56	62	59
30–34	2	3	34	40	46	19	44	57	35	35	10	14	19	25	51	60	62	68
35+	2	3	35	37	38	13	42	56	41	31	8	13	23	18	54	59	66	69
Gravidity																		
First pregnancy	1	2	23	25	30	9	25	41	35	32	7	7	10	8	41	31	28	25
Not first pregnancy	2	3	34	39	41	15	40	59	40	38	11	12	16	17	53	58	61	63

Attendance (%) gives the percentage of women who had attended a women's group among those who recently delivered a baby.

Definition of intervention years per trial: **Mumbai trial**: Years 1–3: October 2006–September 2009. **First Bangladesh trial**: Years 1–3: February 2005–December 2007. **Second Bangladesh tria**l: Year 1: January 2009–December 2009; Year 2: January 2010–December 2010; Year 3: January 2011–June 2011. **Rural India trial**: Year 1: August 2005–July 2006; Year 2: August 2006–July 2007; Year 3: August 2007–July 2008. **Nepal-Makwanpur trial**: Year 1: November 2001–October 2002; Year 2: November 2002–October 2003. **Nepal-Dhanusha trial**: Year 1: 14 April 2007–12 April 2008; Year 2: 13 April 2008–13 April 2009; Year 3: 14 April 2009–13 April 2010; Year 4: 14 April 2010–13 April 2011. **Malawi trial**: Year 1: May 2005–January 2006; Year 2: February 2006–January 2007; Year 3: February 2007–January 2008; Year 4: February 2008–January 2009. Year 1 includes 9 month run-in period in which the groups were formed but not all were fully established. These 9 months were excluded from the original trial paper. These 9 months were included in our analysis as several other sites also experienced some set-up issues in their first year, with late start or disruptions of women's groups in some clusters.

**Table 4:** Socioeconomic and sociodemographic differences in women’s group attendance (ORs), urban India trial

	Mumbai, urban India trial	
	OR	95% CI
Maternal education, years schooling		
Never went to school	1	
1–4	1.39	(0.61 to 3.19)
5–10	1.07	(0.65 to 1.76)
11+	0.92	(0.44 to 1.93)
p Value*	*0.834*	
Household economic status		
Poorest	1	
Next poor	1.37	(0.74 to 2.52)
Middle	1.28	(0.70 to 2.34)
Next rich	0.93	(0.48 to 1.80)
Rich	1.19	(0.62 to 2.29)
p Value*	*0.672*	
Maternal age, years		
<20	1	
20–24	0.92	(0.42 to 1.99)
25–29	1.61	(0.75 to 3.48)
30–34	1.58	(0.65 to 3.84)
35+	2.09	(0.66 to 6.64)
p Value*	*0.063*	
Gravidity		
First pregnancy	1	
Not first pregnancy	1.53	(1.00 to 2.34)
p Value*	*0.051*	

*Combined Wald test for null hypothesis that overall effect of social position was equal to 1 (no effect).

**Table 5:** Socioeconomic and sociodemographic differences in women's group attendance (ORs), per intervention year, two trials in Bangladesh

			2nd Bangladesh trial						
	1st Bangladesh trial		Year 1		Year 2		Year 3		
	OR	95% CI	OR	95% CI	OR	95% CI	OR	95% CI	p Value SP×year*
Maternal education, years schooling									
Never went to school	1		1		1		1		
1–4	1.07	(0.86 to 1.32)	1.08	(0.91 to 1.29)	0.95	(0.80 to 1.13)	1.12	(0.86 to 1.45)	
5–10	0.69	(0.54 to 0.89)	0.74	(0.62 to 0.88)	0.76	(0.64 to 0.90)	0.74	(0.57 to 0.96)	
11+	0.79	(0.44 to 1.44)	0.26	(0.16 to 0.44)	0.39	(0.25 to 0.60)	0.31	(0.15 to 0.62)	
p Value†	*0.004*		*0.000*		*0.000*		*0.000*		*0.516*
Household economic status									
Poorest	1		1		1		1		
Next poor	0.9	(0.67 to 1.20)	0.76	(0.62 to 0.92)	0.86	(0.71 to 1.03)	0.8	(0.59 to 1.07)	
Middle	0.86	(0.63 to 1.16)	0.76	(0.63 to 0.92)	0.95	(0.78 to 1.15)	0.73	(0.53 to 1.02)	
Next rich	0.93	(0.69 to 1.26)	0.67	(0.55 to 0.81)	0.84	(0.69 to 1.01)	0.9	(0.66 to 1.22)	
Rich	0.72	(0.51 to 1.02)	0.54	(0.44 to 0.67)	0.58	(0.47 to 0.72)	0.53	(0.37 to 0.75)	
p Value†	*0.406*		*0.000*		*0.000*		*0.003*		*0.426*
Maternal age, years									
<20	1		1		1		1		
20–24	1.04	(0.78 to 1.39)	1.2	(0.98 to 1.47)	1.37	(1.13 to 1.67)	1.24	(0.94 to 1.65)	
25–29	1.52	(1.14 to 2.03)	1.57	(1.27 to 1.94)	1.81	(1.48 to 2.21)	1.34	(1.00 to 1.80)	
30–34	1.39	(0.98 to 1.98)	1.58	(1.23 to 2.02)	1.85	(1.46 to 2.34)	1.78	(1.26 to 2.51)	
35+	1.2	(0.77 to 1.85)	1.61	(1.16 to 2.23)	1.66	(1.22 to 2.25)	1.29	(0.82 to 2.03)	
p Value†	*0.005*		*0.000*		*0.000*		*0.025*		*0.702*
Gravidity									
First pregnancy	1		1		1		1		
Not first pregnancy	1.75	(1.40 to 2.17)	1.74	(1.47 to 1.95)	1.92	(1.67 to 2.21)	1.63	(1.33 to 2.00)	
p Value†	*0.000*		*0.000*		*0.000*		*0.000*		*0.377*

*Combined Wald test, to test if the effect of social position on attendance had changed statistically significantly over the years.

†Combined Wald test for null hypothesis that overall effect of social position was equal to 1 (no effect).

**Table 6:** Socioeconomic and sociodemographic differences in women’s group attendance (ORs), per intervention year, rural India trial

	Rural India trial						
	Year 1		Year 2		Year 3		
	OR	95% CI	OR	95% CI	OR	95% CI	p Value SP×year*
Maternal education, years schooling							
Never went to school	1		1		1		
1–4	1.40	(0.92 to 2.14)	0.90	(0.63 to 1.28)	1.22	(0.86 to 1.74)	
5–10	1.20	(0.93 to 1.54)	0.97	(0.79 to 1.19)	0.92	(0.75 to 1.12)	
11+	1.17	(0.50 to 2.76)	0.72	(0.38 to 1.37)	0.34	(0.20 to 0.57)	
p Value†	*0.264*		*0.715*		*0.000*		*0.062*
Household economic status							
Poorest	1		1		1		
Next poor	1.44	(1.09 to 1.90)	1.36	(1.11 to 1.66)	1.27	(1.03 to 1.56)	
Middle	1.09	(0.74 to 1.61)	1.22	(0.93 to 1.60)	1.44	(1.09 to 1.88)	
Next rich	2.11	(1.41 to 3.16)	1.06	(0.78 to 1.44)	1.00	(0.74 to 1.35)	
Rich	1.50	(0.98 to 2.30)	1.08	(0.78 to 1.51)	0.44	(0.32 to 0.62)	
p Value†	*0.003*		*0.031*		*0.000*		*0.000*
Maternal age, years							
<20	1		1		1		
20–24	1.30	(0.91 to 1.86)	1.36	(1.02 to 1.82)	1.49	(1.13 to 1.95)	
25–29	1.94	(1.37 to 2.75)	2.08	(1.55 to 2.78)	1.80	(1.37 to 2.38)	
30–34	2.12	(1.44 to 3.11)	2.39	(1.74 to 3.28)	1.80	(1.32 to 2.44)	
35+	1.28	(0.78 to 2.10)	2.17	(1.50 to 3.13)	1.74	(1.20 to 2.53)	
p Value†	*0.000*		*0.000*		*0.000*		*0.253*
Gravidity							
First pregnancy	1		1		1		
Not first pregnancy	1.75	(1.37 to 2.24)	2.00	(1.67 to 2.40)	2.06	(1.74 to 2.45)	
p Value†	*0.000*		*0.000*		*0.000*		*0.546*

*Combined Wald test, to test if the effect of social position on attendance had changed statistically significantly over the years.

†Combined Wald test for null hypothesis that overall effect of social position was equal to 1 (no effect).

**Table 7:** Socioeconomic and sociodemographic differences in women's group attendance (ORs), per intervention year, two trials in Nepal

	Nepal-Makwanpur trial					Nepal-Dhanusha trial								
	Year 1		Year 2			Year 1		Year 2		Year 3		Year 4		
	OR	95% CI	OR	95% CI	p Value SP×year*	OR	95% CI	OR	95% CI	OR	95% CI	OR	95% CI	p Value SP×year*
Maternal education, years schooling														
Never went to school	1		1			1		1		1		1		
1–4	1.06	(0.75 to 1.50)	1.13	(0.77 to 1.65)		0.89	(0.62 to 1.28)	1.05	(0.74 to 1.50)	1.15	(0.86 to 1.53)	1.22	(0.93 to 1.61)	
5–10	1.29	(0.96 to 1.74)	1.14	(0.81 to 1.61)		1.04	(0.67 to 1.60)	1.18	(0.78 to 1.77)	1.27	(0.90 to 1.80)	1.07	(0.76 to 1.51)	
11+	–	–	–	–		1.35	(0.77 to 2.39)	1.12	(0.69 to 1.80)	1.19	(0.79 to 1.79)	0.96	(0.66 to 1.40)	
p Value†	*0.242*		*0.647*		*0.824*	*0.661*		*0.846*		*0.395*		*0.525*		*0.907*
Household economic status														
Poorest	1		1			1		1		1		1		
Next poor	1.03	(0.81 to 1.32)	1.35	(1.04 to 1.76)		0.94	(0.71 to 1.26)	0.95	(0.70 to 1.28)	0.98	(0.73 to 1.33)	0.82	(0.57 to 1.16)	
Middle	–	–	–	–		0.63	(0.44 to 0.90)	0.94	(0.68 to 1.31)	1.03	(0.77 to 1.39)	0.94	(0.68 to 1.28)	
Next rich	1.35	(0.94 to 1.93)	1.21	(0.77 to 1.90)		0.71	(0.49 to 1.03)	0.91	(0.65 to 1.28)	1.28	(0.96 to 1.72)	0.96	(0.71 to 1.31)	
Rich	0.85	(0.57 to 1.26)	1.23	(0.77 to 1.98)		0.53	(0.36 to 0.79)	0.69	(0.49 to 0.97)	1.02	(0.75 to 1.37)	0.64	(0.47 to 0.87)	
p Value†	*0.279*		*0.144*		*0.297*	*0.004*		*0.298*		*0.372*		*0.009*		*0.121*
Maternal age, years														
<20	1		1			1		1		1		1		
20–24	1.6	(1.05 to 2.46)	1.36	(0.72 to 2.59)		1.33	(0.96 to 1.84)	1.53	(1.10 to 2.12)	1.22	(0.93 to 1.59)	1.96	(1.43 to 2.68)	
25–29	1.34	(0.86 to 2.09)	1.98	(1.04 to 3.77)		1.95	(1.39 to 2.74)	2.03	(1.45 to 2.85)	1.67	(1.27 to 2.20)	3.38	(2.47 to 4.63)	
30–34	1.17	(0.73 to 1.89)	1.29	(0.65 to 2.53)		1.53	(0.95 to 2.46)	2.23	(1.45 to 3.43)	2.05	(1.41 to 2.96)	4.90	(3.38 to 7.10)	
35+	1.5	(0.94 to 2.41)	1.11	(0.56 to 2.20)		1.24	(0.64 to 2.40)	2.17	(1.25 to 3.79)	2.59	(1.59 to 4.20)	3.28	(1.97 to 5.45)	
p Value†	*0.120*		*0.008*		*0.033*	*0.002*		*0.000*		*0.000*		*0.000*		*0.007*
Gravidity														
First pregnancy	1		1			1		1		1		1		
Not first pregnancy	1.22	(0.89 to 1.66)	1.33	(0.94 to 1.89)		1.59	(1.21 to 2.07)	1.85	(1.21 to 2.07)	1.66	(1.34 to 2.06)	2.54	(2.02 to 3.21)	
p Value†	*0.212*		*0.112*		*0.710*	*0.001*		*0.000*		*0.000*		*0.000*		*0.025*

*Combined Wald test, to test if the effect of social position on attendance had changed statistically significantly over the years. †Combined Wald test for null hypothesis that overall effect of social position was equal to 1 (no effect).

**Table 8:** Socioeconomic and sociodemographic differences in women's group attendance (ORs), per intervention year, trial in Malawi

	Malawi trial								
	Year 1		Year 2		Year 3		Year 4		p Value SP×year*
	OR	95% CI	OR	95% CI	OR	95% CI	OR	95% CI	
Maternal education, years schooling									
Never went to school	1		1		1		1		
1–4	1.22	(0.96 to 1.55)	1.07	(0.85 to 1.35)	0.93	(0.74 to 1.17)	0.93	(0.71 to 1.21)	
5–10	1.12	(0.89 to 1.42)	0.98	(0.78 to 1.22)	0.76	(0.61 to 0.94)	0.80	(0.62 to 1.03)	
11+	0.59	(0.33 to 1.08)	0.89	(0.54 to 1.47)	0.53	(0.31 to 0.91)	0.68	(0.39 to 1.18)	
p Value†	*0.058*		*0.746*		*0.009*		*0.183*		*0.386*
Household economic status									
Poorest	1		1		1		1		
Next poor	1.16	(0.84 to 1.59)	1.23	(0.92 to 1.64)	1.08	(0.80 to 1.45)	1.02	(0.75 to 1.40)	
Middle	1.27	(0.99 to 1.63)	1.23	(0.97 to 1.55)	1.05	(0.83 to 1.32)	1.14	(0.89 to 1.47)	
Next rich	1.17	(0.94 to 1.46)	1.24	(1.01 to 1.51)	0.96	(0.79 to 1.18)	1.09	(0.88 to 1.36)	
Rich	0.80	(0.51 to 1.26)	1.00	(0.67 to 1.50)	0.85	(0.55 to 1.32)	0.60	(0.38 to 0.95)	
p Value†	*0.167*		*0.224*		*0.815*		*0.090*		*0.873*
Maternal age, years									
<20	1		1		1		1		
20–24	1.48	(1.12 to 1.95)	2.48	(1.86 to 3.29)	2.72	(2.06 to 3.58)	2.27	(1.68 to 3.07)	
25–29	1.89	(1.41 to 2.53)	2.98	(2.21 to 4.02)	3.96	(2.96 to 5.30)	3.06	(2.24 to 4.19)	
30–34	1.59	(1.16 to 2.19)	3.40	(2.49 to 4.65)	3.99	(2.92 to 5.44)	4.61	(3.28 to 6.48)	
35+	1.83	(1.31 to 2.54)	3.40	(2.44 to 4.73)	4.88	(3.52 to 6.77)	4.72	(3.34 to 6.69)	
p Value†	*0.000*		*0.000*		*0.000*		*0.000*		*0.000*
Gravidity									
First pregnancy	1		1		1		1		
Not first pregnancy	1.61	(1.28 to 2.02)	3.07	(1.68 to 3.07)	3.87	(3.10 to 4.84)	4.96	(3.83 to 6.41)	
p Value†	*0.000*		*0.000*		*0.000*		*0.000*		*0.000*

*Combined Wald test, to test if the effect of social position on attendance had changed statistically significantly over the years.

†Combined Wald test for null hypothesis that overall effect of social position was equal to 1 (no effect).

There is little evidence to support the hypothesis that the interventions systematically reached higher socioeconomic groups first (in year 1 or when overall levels were low). Where overall attendance was low (Mumbai and first Bangladesh trial), it was at least as high among lower as among higher strata. Elsewhere, in year 1, the interventions reached lower socioeconomic groups to a similar extent as or greater extent than they did higher strata, except in the trial in rural India. Increases in attendance over time were usually at least as strong among lower/middle socioeconomic groups as among higher groups. In rural India, the increase was weaker among the elite, causing a reversal of the association between socioeconomic position and attendance (p for changed effect <0.001 for economic status, 0.062 for education).

The associations between socioeconomic position and attendance remained largely unchanged after adjusting for sociodemographic position (results not shown). Only in some instances (Dhanusha-Nepal Y3-4, rural-India Y1) did the more highly educated show higher attendance levels after adjusting for age or gravidity. The strong effect of sociodemographic position on attendance was not explained by socioeconomic position. Furthermore, gravidity had strong independent effects after adjusting for age everywhere except Makwanpur, Nepal. The effects of age were largely explained by gravidity; substantial age effects only remained in some cases.

### Qualitative findings

#### Why were socioeconomic differences in uptake small?

Even when the interventions did not systematically target women from lower social strata, facilitators were keen to involve them and worked hard to motivate them to attend: “She has worked very hard. She goes door-to-door once, twice and three times to invite women to join the group” (non-attender, Bangladesh). The facilitators encouraged all women to attend: “she comes and convinces, saying ‘let's go. If you go to the meeting then it will be good for us and for the village. You will know about the things that you did not know before’” (non-attender, Dhanusha, Nepal). The trial in rural India explicitly targeted lower socioeconomic groups, primarily by convening meetings in clusters of houses where poorer tribal people lived. Many participants felt the groups were not exclusive to a socioeconomic group and membership was open: “The women's group is for everyone. Anyone can come to learn something” (attender, Makwanpur, Nepal); “Even a woman who had gone abroad in an aeroplane (a wealthy woman), her baby died because she didn't have health information” (attender, rural India). All trials used simple discussion tools and approaches that interested and engaged women from all strata. “The women understand everything because of the way we say things. They enjoy and want to participate and be a part of the group. If we tell them stories, generally, women do understand very well, but when we say the same thing with picture cards then they like it” (facilitator, rural India). Attenders told us, “I go to a lot of meetings. They explain things very nicely” (key informant, Bangladesh). “We had fun. We saw the game played by others, and saw pictures which helped broaden our mind” (attender, Dhanusha, Nepal). The meetings were close to women's homes and at convenient times, enabling attendance: “If the place is nearby, everyone comes to the meeting” (attender, rural India); “the meeting used to happen in our house… it was easy to go to the group” (attender, Mumbai). Many groups ran savings or emergency funds and were supportive in arranging transport at the time of delivery. Participants believed that attendance of the elite was lower because they did not need knowledge and financial or social support from groups, and because some of them felt the groups were beneath them: “Rich people think their status will be demeaned if they come together with the poor in the groups” (facilitator, Malawi). The higher attendance among better-off women in year 1 of the rural India trial was thought to have diminished in subsequent years because of their ability to grasp information more quickly: “After a while they knew everything, and then they did not come. Later, others came to learn new things” (non-attender, rural India).

#### Why were sociodemographic differences in uptake large?

Participants were not surprised by the low attendance among young primigravid women. In all sites, they were less likely to attend because they were uncomfortable discussing reproductive health issues in front of older, more experienced women. Some respondents thought that the groups were not relevant or appropriate for women who were not yet pregnant or were childless. In Malawi, group members reportedly excluded younger women when they attended: “The old ones thought that the group had nothing to do with the young women. No wonder they stopped coming” (attender, Malawi). Young women themselves did not feel comfortable in the group, and non-attenders told us, “when (young women) went to the group they found out that it was for mature women, so they stopped coming…” (non-attender, Malawi). In the Asian sites, group members understood why attendance was low among young women, but did not actively exclude them in the same way. Families in all Asian sites often believed that young women were vulnerable to evil spirits and that pregnancies might be affected by the complications discussed in the group: “some husbands do not want to let their wives out of the house. They fear that they may be possessed by evil spirits” (decisionmaker, Bangladesh). Asian women were particularly affected by the social taboo that newly married women should not go outside their husbands’ house without permission. Young women were vulnerable to getting a bad reputation, which would harm the family: “because of the thinking that a new daughter-in-law should not roam around the village, they told me not to go” (non-attender, Dhanusha, Nepal). Families also feared that a newlywed would gossip about her ‘new’ household, and that she might learn bad habits from other community members: “in-laws do not allow newly married women to go to the group meeting because they think that if they come to the meeting other women may teach them something else and they will run away from their home” (facilitator, rural India). Facilitators told us, “some say we are making women too clever through the group. Women won't respect men anymore” (Bangladesh). The household would be upset and ashamed if internal conflict was caused by their daughter-in-law becoming more active. The family would also feel socially humiliated by this indication that they were not looking after her: “people will say, ‘she only arrived in the family yesterday, and already she is going to groups’” (non-attender, Dhanusha, Nepal). Primigravid women who had supportive families, or fewer restrictions, were able to come to the group: “my mother-in-law wasn't there, that is why I didn't feel scared to sit in the meeting” (attender, Mumbai). Families who believed it was important for young women to learn encouraged them to attend. To include young primigravid women, participants suggested working with families to convince them to send their daughters-in-law to groups. Participants also believed that generally increasing awareness about the objectives of the group, and ensuring buy-in from important people in the community, would help families understand the benefits of groups and dispel concerns.

## Discussion

Community-based participatory women's groups to improve newborn survival reach all socioeconomic strata by-and-large equally, except for a lower uptake among the socioeconomic elite. By contrast, sociodemographic differences were large, with a much lower intervention uptake among socially and biologically more vulnerable young and primigravid women; this was not just a South Asian phenomenon: the same pattern, perhaps stronger, was observed in the African trial.

We used data from surveillance systems in which full populations were prospectively followed-up. Non-response rates were very low. Qualitative data collection was complicated for trials conducted several years previously, particularly in Mumbai, where attendance had been low and many women had moved since the trial. We overcame recall difficulties by using a ‘think back’ methodology.

Our findings contrast with a body of literature showing that interventions reach higher socioeconomic groups first and to a greater extent, and that uptake among lower strata only increases once the better-off cannot improve further.^8^ ^10^ ^15^ ^36^ Furthermore, some studies suggest that community-based interventions can reinforce social hierarchies, while we show that a community-based approach can be socioeconomically inclusive, reaching those who need the intervention the most. The extent of differential intervention uptake is arguably associated with the extent of social stratification, with more equitable uptake in more equitable societies. Yet, the large rural and urban communities under study (>2 million population) were, as in most LMICs, not homogeneously poor. They exhibited important social hierarchies according to wealth, literacy and caste, and large inequalities in intervention uptake have been reported for such populations.^37–39^

Our qualitative findings show that local facilitators were key to equitable intervention reach, concurring with findings of others that quality of facilitators is an important success factor in community-based projects.^19^ The facilitators were not dissimilar from the group members and were respected. While they were often better educated and more able to move around the community, they resembled the group members in terms of where they were from, their family situation and their understanding of the local context. They promoted inclusivity by encouraging poorer women to attend. The intervention design was also important: attractive and inclusive participatory tools and approaches presented information in forms that enabled less-educated women to understand. Furthermore, the groups were accessible, arranged according to women's convenience. The intervention met a need—an information and discussion gap around reproductive health—that all women were interested in engaging with. Importantly, groups were perceived to be for all social strata, or even for women with greater need.

The lower uptake of the intervention among young primigravid women is noteworthy, given that in some of the trials they were particularly encouraged to attend.^40^ ^41^ Moreover, the intervention sought to improve maternal and newborn health, and 16 million births worldwide are to women aged 15–19 years.^42^ While women's oppression is a well-known phenomenon in South Asia, the intersection of gender, age and gravidity, with young primigravid women having less freedom to participate in community-based reproductive health interventions, was also important in the African site. Perceptions that it is inappropriate or inauspicious for young women to discuss reproductive health reinforce young women's embarrassment in discussing these issues, especially with older, more experienced women. Social taboos and family control restrict young women's movement outside the home and affect access to groups in the South Asian context. Awareness campaigns may increase the social acceptability of reproductive health discussions, in order for young women to feel less embarrassed. Many group members had initiated contact with young pregnant women, which may be beneficial in areas where social taboos are stronger. Some participants suggested separate groups for young women, but this may make it more difficult for them to attend when families are suspicious. The effectiveness of exclusive young women's groups in improving reproductive health should be explored.

## Conclusion

Community-based approaches through women's groups can address the exclusion of lower socioeconomic groups from health interventions and can help to reach every newborn. The following factors facilitate equitable intervention uptake: local facilitators who encourage all women to attend, attractive approaches that are accessible to lower socioeconomic groups, and ensuring that the intervention meets a perceived need and is arranged at the participants’ convenience. The lower uptake of community-based newborn health interventions among young primigravid women deserves specific research and policy attention, since they may be excluded if generally inclusive approaches are taken. Specific efforts are needed to increase the social acceptability of including them in reproductive health discussions.

**What is already known on this subject**

- Socioeconomic inequalities in maternal and newborn health are large, and health interventions rarely reach the most in need. Community-based interventions to improve newborn health are becoming increasingly popular, but they run the risk of elite capture, in which the locally powerful influence interventions to their own benefit. Little is known about how to effectively reach the poor and otherwise vulnerable groups.

**What this study adds**

- Community-based women's groups can address the exclusion of lower social strata from health interventions. Equitable uptake is promoted when facilitators organise the intervention at the convenience of poor participants, use engaging approaches that are easily understandable for the less educated and actively encourage all socioeconomic groups to attend. Young primigravid women, who are biologically and socially more vulnerable, run the risk of being excluded from community-based interventions due to social taboos, and addressing this deserves specific policy and research attention.
